# The COVID-19 Puzzle

**DOI:** 10.6026/97320630016418

**Published:** 2020-05-31

**Authors:** Leszek Konieczny, Irena Roterman

**Affiliations:** 1 Jagiellonian University - Medical College, Krakow, Poland

**Keywords:** COVID-19, alcohol, Mediterranean diet, misfolded proteins, microbiom

## Abstract

Viral diseases have affected humans since the dawn of humanity. Smallpox-now eradicated by vaccinations-serves as a particularly poignant example. More recently, the Spanish flu outbreak claimed a heavy toll in the early 20th century. The ongoing coronavirus pandemic appears no less threatening. The possible reason of highly variable course of disease is discussed.

## Views:

Vaccinations presented a breakthrough in the treatment of viral diseases. Other promising drugs include inhibitors of viral enzymes. Unlike bacteria, viruses are not, strictly speaking, alive. They are packets of information capable of invading and exiting cells. The host cell is burdened with synthesizing and replicating the virus, whose capability for penetrating cells determines its potency. From the epidemiological perspective, an important factor affecting the spread of the virus is the proximity between infection targets. Viruses capable of droplet transmission are particularly dangerous. Bacteria are evolutionarily ancient entities that have long been targeted by certain classes of viruses (known as phages). As a result, some bacteria have evolved methods to combat hostile carriers of information. The bacterial genome includes a repository of viral genetic sequences-effectively a "catalogue" of known viruses. When a virus invades, the bacterium responds by synthesizing (based on its stored DNA) a complementary RNA matrix attached to a nuclease-a DNA-degrading enzyme. This complex is capable of recognizing the viral genome and cleaving it, disrupting the invasion [1,2]. Eukaryotic organisms, such as humans and animals, have evolved other methods of combating viruses: antibodies, which can destroy attackers and prevent further attacks. However, such mechanisms require some time to kick in and initiate an immune response, particularly when dealing with a novel virus which they haven't encountered before.

The coronavirus responsible for the ongoing pandemic is spreading very rapidly. Its effects appear to depend on the carrier's age: many people, particularly young ones, remain asymptomatic, while older individuals frequently develop serious-even life-threatening-symptoms. The disease also appears to affect men more frequently than women, while its prevalence varies by region. Of course, social measures may either promote or hamper the spread of the virus; however, this factor alone does not fully explain the observed dynamics of the disease. A convincing argument can be made for the existence of other factors, such as varying susceptibility to the virus. Variable susceptibility to infection may be a consequence of the general state of health, which, of course, depends on the individual's age and co-morbidities-particularly metabolic ones (e.g. diabetes). Nevertheless, the observed profile of the disease suggests that other factors are at play and may determine whether a given organism can successfully defend itself against the virus. Since the virus preferentially attacks the lungs, we can point to tobacco smoking as a factor, which promotes infection by damaging lung tissue. However, there are other factors as well, one of which is alcohol consumption [3-5]. Alcohol as a solvent is inherently alien substance-from the point of view of cells-and, of course, toxic in itself. Even at low concentrations it may alter the structural properties of water, which can have an effect on solvated proteins and cellular membranes.

One mechanism which exhibits clear susceptibility to external disruption (including the presence of unusual substances) is protein folding. Folding is usually understood as the process by which a protein attains its tertiary structure. This structure is strongly dependent on environmental conditions, and also on changes in the protein’s environment [6- 10]. Cells have developed mechanisms, which prevent aggregation of improperly folded proteins whose presence may endanger each cell and, consequently, the organism as a whole. For example, evolution has equipped cells with the so-called UPR (Unfolding Protein Response) process. This is a mechanism by which special "watchdog" proteins recognize errors in the folding process and fix them, or-when that is no longer possible-trigger cell death. The resulting balance between threats and defense mechanisms is nevertheless precarious, and may be destroyed by external stimuli-for example, by excess presence of alcohol. Organisms can tolerate low levels of alcohol and reverse the damage it causes. If, however, alcohol concentration rises to a point where it can be detected by a probe, or-even more worryingly-smelled on the subject's breath, the situation is no longer benign, and damage to liver and lung tissue may ensue. It should be noted that alcohol itself is not as toxic as its primary metabolite-acetaldehyde [11- 15].

Acetaldehyde readily reacts with amines, including with guanine residues present in nucleic acids, which-when placed in close proximity-may bind to each other. The resulting complexes are usually unstable and fall apart quickly. Even guanine pairs (relatively stable in comparison) are generally repaired in liver tissue by the appropriate enzymes. However, if a mutation knocks out reparatory mechanisms-such as in the case of Fanconi anemia-the damage incurred by DNA may become severe and lead, among others, to carcinogenesis. Notably, ε-amine groups in lysine residues are damaged by the aldehyde, and this type of damage is believed to be long lasting. Another example of aldehydes acting upon proteins involves damage to microtubules, which play an important role in ensuring structural stability of the cell. Genetic polymorphism undoubtedly results in individual variability, including well-known differences in alcohol tolerance. Societies used to consuming large quantities of alcohol-usually in small doses-are characterized by specific public health traits, such as those attributable to resveratrol. Alcohol is also increasingly implicated in a variety of pathological conditions, including cancer. Regardless of specifics, its effect upon the organism- including lung tissue-is undoubtedly significant. Identifying links between alcohol use and the kinetics of viral infection remains a speculative endeavor. Further information may be provided by statistical studies involving a large quantity of individual habits and cases.

Professional literature also suggests a possible way by which alcohol may affect the reactivity of the organism and its interaction with the environment. This mechanism refers to the so-called bacteriome, i.e. our hybrid bacterial ecosystem [16-19]. Bacteria with which we interact outnumber the cells in our bodies by roughly an order of magnitude. This enormous population-trillions of bacteria-mainly inhabits the digestive trait, but some bacteria are also found in skin and lung tissue. Recent research has increasingly linked the composition of individual bacteriomes with those of the corresponding organisms. Bacteriomes are known to be affected by diet, personal hygiene, tobacco smoking, alcohol consumption etc. Dietary habits vary from nation to nation. In some regions, frequent consumption of alcoholic beverages has a profound effect on bacterial populations-with predictable consequences. The microbiome also appears to affect immune system function, with imbalances usually having a detrimental effect. This balance is rather precarious-a weak immune reaction is clearly undesirable, but an overreaction leading to severe inflammation and necrosis, is also dangerous. Age appears to correlate with the severity of viral infection. This is clearly associated with impairment of the immune system, but may also be due to co-morbidities, which frequently occur at an advanced age [20,21]. The rapid onset and spread of the coronavirus pandemic raises questions, which we cannot yet answer with certainty. Among the most important of those is why the course of the disease exhibits such dramatic variability-with some subjects exhibiting severe symptoms while others are barely affected at all. It can be hoped that ongoing research will eventually cast some light on these issues, thereby preparing us to better cope with future viral diseases. The approach based on the influence of alcohol on the protein folding process is shown in [22].

## References

[1] Kim JS (2018). Science.

[2] Dolgin E (2019). Nature.

[3] Yin SJ (1992). Biochem Genet..

[4] Gallina I, Duxin JP (2020). Nature.

[5] Rintala J (2000). Alcohol Alkohol..

[6] Halliwell B (2006). J Neurochem.

[7] Ding Q (2003). J Neurochem.

[8] Mimnaugh EG (1997). Biochemistry..

[9] Johnson JR (2016). Genetics..

[10] Thomas PJ (1995). Trends Biochem Sci..

[11] Nicholls R (1992). Int J Biochem.

[12] Moises O Fiesco-Roa (2019). Blood Rev.

[13] Langevin F (2011). Nature.

[14] Tuma DJ (1991). Alcohol Alcohol Suppl..

[15] Sonohara Y (2019). Sci Rep..

[16] Helmink BA (2019). Nat Med..

[17] Ajami NJ, Wargo JA (2020). Nature.

[18] Pulendran B (2019). Science..

[19] Zitvogel L, Kroemer G (2019). Science..

[20] O'Toole PW, Jeffery IB (2015). Science.

[21] Martinez-Vicente M (2005). Exp Gerontol.

